# Integrating Multicellular Systems: Physiological Control and Degrees of Biological Individuality

**DOI:** 10.1007/s10441-023-09476-4

**Published:** 2023-12-27

**Authors:** Leonardo Bich

**Affiliations:** Department of Philosophy, IAS-Research Centre for Life, Mind and Society, University of the Basque Country (UPV/EHU), Avenida de Tolosa 70, Donostia-San Sebastian, 20018 Spain

**Keywords:** Physiology, Organization, Biofilms, Animals, Vascularization, Nervous System

## Abstract

This paper focuses on physiological integration in multicellular systems, a notion often associated with biological individuality, but which has not received enough attention and needs a thorough theoretical treatment. Broadly speaking, physiological integration consists in how different components come together into a cohesive unit in which they are dependent on one another for their existence and activity. This paper argues that physiological integration can be understood by considering how the components of a biological multicellular system are controlled and coordinated in such a way that their activities can contribute to the maintenance of the system. The main implication of this perspective is that different ways of controlling their parts may give rise to multicellular organizations with different degrees of integration. After defining control, this paper analyses how control is realized in two examples of multicellular systems located at different ends of the spectrum of multicellularity: biofilms and animals. It focuses on differences in control ranges, and it argues that a high degree of integration implies control exerted at both medium and long ranges, and that insofar as biofilms lack long-range control (relative to their size) they can be considered as less integrated than other multicellular systems. It then discusses the implication of this account for the debate on physiological individuality and the idea that degrees of physiological integration imply degrees of individuality.

## Introduction

Multicellularity is a common phenomenon that cuts across all the domains of life, from bacteria to plants and animals. It is thought to have originated independently in both prokaryotes and eukaryotes at least 25 times (Grosberg and Strathmann 2007).^1^ Multicellular systems are cohesive entities composed of cells and extracellular components such as the extracellular matrix (ECM), or the extracellular polymeric substances (EPS) in the case of biofilms. Their organization takes many different forms. They can span across a wide range of examples from bacterial biofilms to animals, plants and fungi. They include also limit cases of minimally organized facultative multicellular systems such as, among others, the Choanoflagellates of the species *Salpingoeca rosetta*, eukaryotic organisms which can live as free unicellular systems but, in response to diverse environmental cues, form simple chains or spherical colonies kept together by cytoplasmatic bridges and extracellular matrix (Larson et al. 2020).

The extant variety of possible multicellular systems raises the question of how to characterize, compare and distinguish them. This paper aims to take some first steps towards that end by focusing on their organization and, more specifically, on the degree of physiological integration that different multicellular organizations can achieve. In general, the integration of a biological system can be defined in terms of the degree of mutual dependence between its components. From the physiological point of view, it consists in how different components come together into a cohesive unit in which they are dependent on one another for their own production, maintenance, and activity. The core idea of this paper is that the notion of control plays a central role in achieving integration, and it needs to be analyzed in detail. Specifically, on this view the physiological cohesiveness we ascribe to multicellular systems can be understood by considering: (1) how the components of a biological multicellular system are controlled, and their activities coordinated, and (2) that they do it in such a way that they can contribute collectively to the maintenance of the system. On this account, the different ways of controlling their parts can give rise to different types of multicellular organizations characterized by different degrees of integration.

This type of analysis has implications for the notion of biological individuality. The concept of integration is often deployed in accounts of biological individuality, and characterized in terms of cohesion, interdependence of parts, and division of labor (Godfrey-Smith 2013; Skillings 2016). The philosophical debate about biological individuality has usually focused on the definition and characterization of *evolutionary* individuals and has helped clarify the discussion about units of selection and the requirements for evolution by natural selection. The philosophical discussions have paid less attention to other, non-evolutionary based accounts (see Lidgard and Nyhart 2017), among them those that appeal to physiology as an alternative way to ground biological individuality, and which are still missing a thorough theoretical treatment. In this context, the notion of physiological integration is supposed to play a central role (Pradeu 2010). However, as argued by Militello et al.(2021) it has been mostly used as an *explanans* and has not been characterized in detail.

This paper pursues an approach to these issues based on the organizational account, which has provided some basic conceptual tools to understand integration and individuality (Moreno and Mossio 2015; Arnellos et al. 2014; Bich et al. 2019; Militello et al. 2021; Bich and Bechtel 2022). From this perspective, physiological integration in a biological system can be characterized as the degree of interdependence between the subsystems that are necessary to realize and maintain the system that harbors them. It entails a mutual dependence between parts but requires much more than that. In living systems, different parts or groups of parts provide different and specific contributions to the functioning and maintenance of the system. Harboring components capable of playing different functional tasks is a fundamental requirement for division of labor. However, a cohesive integration between these different tasks is only achieved when those different activities are orchestrated so that they collectively contribute to the maintenance of the system. To so do and carry out the activities required to maintain itself while avoiding internal conflicts, a biological system needs to control its parts and coordinate them. Control is therefore fundamental to establish mutual dependence between parts and achieve integration. Such control is manifest within living cells, where multiple regulatory control subsystems coordinate several types of kinetic (enzymes), spatial (compartments and membranes) and template (genetic sequences) components into one coherent self-maintaining and self-producing system (Bich et al. 2016; Ruiz-Mirazo et al. 2017).

This paper addresses the question of the physiological integration in multicellular systems by focusing on how they realize control. It discusses the role played by differences in spatial ranges of control of the activities of cells, by analyzing the effects of short, medium and long-range control. 2 Section 2 provides a general characterization of control from an organizational perspective. It adopts a notion of control first proposed by Howard Pattee in terms of constraints (Pattee 1972) and recently developed in the organizational framework (Bich et al. 2016) and beyond (Winning and Bechtel 2018). This conceptual approach has been primarily developed by focusing on the organization of unicellular systems. Applying it to the study of multicellularity, however, presents additional challenges, related to how cells can live together in higher-order biological systems. Sections 3 and 4 examine how control is realized in two types of multicellular systems, biofilms and animals, located at opposite ends of the spectrum of multicellular organizations. This examination reveals that, to a first approximation, a high degree of integration requires control exerted at all short, medium, and long ranges. Section 5 discusses the roles of different ranges of control and addresses their implications for the individuality debate. It shows that integration comes in degrees and suggests that so too should notions of physiological individuality based on it.

## Control in Biological Systems

The organizational framework characterizes biological systems as organized in such a way that they realize metabolic self-production and self-maintenance. Their components exchange matter and energy with their environment, realizing a network that supports their existence and activity (Moreno and Mossio 2015). As discussed by Levy and Bechtel (2013: p. 243) an organization “involves an internal division of labor whereby different components perform different causal roles”. Systems that do not involve differential causal roles for their components are not organized. The specificity of a living system as characterized by the organizational framework is that its components are produced within the system, and that the different activities of these components contribute to the maintenance of the other components and of the living system as a whole. These activities cannot all be realized simultaneously due to spatial and energetic limitations; hence those needed in the current situation need to be selected. Moreover, some parts or subsystems may work differently and with different requirements. These are not always compatible, and their operations need to be modulated in such a way that they can jointly contribute to the maintenance of the system while avoiding potential conflicts. In terms of physiology, the integration between the different functional tasks is only achieved when the differentiation of activities is coordinated at the system level so that the differentiated components contribute to the maintenance of the system. To achieve this end and maintain the existence of the components and the overall network, living systems need to control the activity of these components so that each operates when needed and compatibly with the state of the system and the activities of the other components^3^. This physiological regime is in turn the result of a developmental process, which also requires the fine tuning of the activities of components to bring forth functional changes at every step, while maintaining the system viable (see Bich and Skillings 2023; Montévil and Soto 2023).

Control is generally understood in biology as the capability to actively modify the dynamics of a system toward certain states in a given situation (Rosen 1970). Metabolic control, for example, is characterized as a modification of the state of metabolism in response to signals (Fell 1997). Control implies an asymmetric interaction: there is a controller which acts upon a controlled process, component, or subsystem. A self-controlling system, such as a biological one, should be able to employ different control components to modify its internal processes and the activities of its parts in response to variation in internal and external conditions, rather than only passively undergoing change driven by perturbations.

According to the organizational framework, the capability of living systems to steer or harness a process, or to modulate the activity of their constituents, can be understood in terms of constraints: structures that act as local boundary conditions that enable specific processes and activities. A constraint C is a material structure that harnesses a process P by reducing its degrees of freedom so that:

(1) at a time scale characteristic of P, C is locally unaffected by P;

(2) at this time scale C exerts a causal role on P, i.e. there is some observable difference between free P, and P under the influence of C (Mossio et al. 2013).

Constraints exert a distinctive causal power, which consists in limiting the range of possible outcomes (degrees of freedom) of a process, thus making a specific outcome possible. A constraint is not part of the process it modifies, and it is stable during the time scale in which the process takes place. Through its activity, it canalizes a process toward outcomes that otherwise would be extremely improbable or practically impossible. An example of constraint is a pipe harnessing the flux of water from a pond to a tank located at a given distance across some hills and a valley: a process which would not occur, or not as efficiently, by diffusion alone. In this case the constraint (i.e., the pipe) reduces the degrees of freedom of processes or of collections of elements (the possible direction of movement of the molecules of water) in such a way that they exhibit specific behaviors (like molecules flowing in the same direction). This constrained behavior can be used to perform some coherent activity in the context of the system (such as water filling a tank). A typical biological example is the activity of an enzyme, which catalyzes a reaction without being directly affected by it. The distinctive character of biological systems is that they are capable of generating some of the (internal) constraints that are necessary for their own functioning and that harness the dynamical behavior of their components so that they can maintain themselves in far from equilibrium conditions, an idea captured by the notion of ‘closure of constraints’ (Montévil and Mossio 2015).

Most constraints are realized by structures which statically reduce degrees of freedom of the process they canalize. It is the case of a pipe or of a lipidic semipermeable membrane. This may be sufficient to enable and harness a process in most basic scenarios. Control, however, implies something more: i.e., the capability of modulating and coordinating the activities of the components of a system towards a certain behavior or goal state. This cannot be achieved by means of static structural constraints. As pointed out by Pattee (1972), control requires a special type of constraint. It requires the presence of dynamic constraints that actively select between the degrees of freedom available in a process or a component. This can be achieved for example when a constraint enables or inhibits a process in the presence of signal molecules or of specific conditions in its surroundings. By operating in this way, control constraints do not reduce degrees of freedom once and for all. Instead, they are sensitive to the state of the system or the environment, and they dynamically modulate the controlled process or the behavior of other constraints accordingly.^4^

In biological systems the activities of control constraints play a regulatory role as they contribute to the maintenance of the organisms that produce and maintain those very constraints (Bich et al. 2016). A basic example is a kinase protein, which changes its activation status on the basis of the interaction with a ligand or a signal molecule at a different site than the effector site. Based on such interaction, it phosphorylates other proteins thus modifying (inhibiting, activating, modulating) their activities. Within cells, through the coordinated activity of organized constraints such as proteins and supramolecular complexes, control is dynamically exerted upon those components and processes involved in the flow of matter and energy necessary to build the system, run its internal processes, and produce basic behaviors such as movement.

The specificity of multicellular systems is that, while harnessing and modulating thermodynamic processes to sustain their own metabolism, they also exert control upon the activity of groups of cells that constitute their tissues and organs. For these cells to contribute to the maintenance of the system and work as a cohesive entity, a multicellular system requires some internal differentiation, the basic requirement for division of labor. Internal differentiation depends on the presence of components that contribute in different ways to the realization of the system, such as different types of cells and an extracellular matrix (ECM). Through differentiation multicellular systems become, in principle, capable of harboring components that have different functional roles. Integration between these different tasks is achieved when functions are coordinated such that the differentiated components actively contribute to the maintenance of the system. They do so while their activities are being activated, inhibited, or modulated at different moments in time depending on the state of the system. Integration requires control.

Understanding control at the multicellular level presents some challenges. What is controlled and organized is, among other things, the activity of cells or groups of cells, with the aim to avoid conflict between them and make them mutually sustaining. Control can be achieved for example by inhibiting some of their degrees of freedom while enabling specific behaviors, such as in the case of cell differentiation. Importantly, cells are autonomous agents.^5^ They proliferate and move in their environment unless these capabilities are controlled (Soto and Sonnenschein 2011). Cells in multicellular systems cannot be allowed to proliferate at any time. Cell division is mostly inhibited and is activated in specific moments for specific cells. Mobility is inhibited for most cells, with the exception for example of immune cells, but migration of groups of cells can be activated in specific moments during development. Multicellular control is not limited to differentiation, mobility, and proliferation, but it is also exerted on most intercellular activities such as cellular communication and on the internal physiology of cells, for example by triggering gene transcription.

As argued by Bich et al. (2019), an important question concerns what components play the role of controllers in multicellular systems. While most accounts of multicellularity focus only on cells as controllers, cells are not necessarily the only components that behave like dynamical control constraints. Failing to acknowledge that may preclude the development of an understanding of how cohesive multicellular organizations are realized and maintained. It has been argued that the extracellular matrix (ECM) plays a crucial role as controller in many multicellular systems (Bich et al. 2019), with priority with respect to cell-to-cell interactions (Guilak et al. 2009; Rozario and DeSimone 2010). The matrix is a network of several types of proteins and carbohydrates. In eukaryotes it may include collagen, enzymes, glycoproteins, proteoglycans, growth factors, etc. It functionally acts as a control constraint in multicellular systems. Control is exerted by the matrix because it: (1) realizes dynamic three-dimensional structures that are sensitive to the state of the system or a specific tissue, and (2) depending on what they sense, they dynamically constrain, that is, they actively select between possible states of individual cells or groups of cells, such as proliferation and mobility, and different physiological activities. For example, mechanical forces and molecular interactions can alter the functional domains of proteins embedded in the matrix network, so changing their activation state. In turn, the activated proteins can control cell behavior by binding to specific membrane receptors and triggering intracellular signaling processes that activate gene transcription (Halder et al. 2012).

Control is a specific type of causal relation. It involves constraints, but of a special type: dynamical ones that are sensitive to their surroundings and modify the activities of other constraints accordingly. Moreover, by coordinating the activities of different parts in such a way as to maintain the system, control realizes physiological integration. However, control is not performed uniformly, and there may be many ways to instantiate it. The rest of this paper focuses on different ways the differentiated parts of a system are coordinated by control constraints so as to achieve integration. One of the challenges of multicellular organizations is to control the activities of ensembles of cells across different distances in systems of very different sizes, especially in those with increasingly larger bodies and numbers of components. It follows that one useful way to look at the relation between different ways of exerting control and different types or degrees of physiological integration in multicellular systems is in terms of control ranges. One can make an initial broad distinction between three ranges of control: short, medium, and long. Short-range control is mostly exerted through cell-to-cell interactions. Medium-range control is exerted by the extracellular matrix upon cells or groups of cells. Long-range control is enabled primarily by vascularization, but also by the nervous system. It connects distant parts of the system and potentially affects a higher number of them. The next two sections address how differences in the range of control contribute to the overall integration of different types of multicellular systems.

## Biofilms

Biofilms are prokaryotic multicellular systems realized by the association of bacteria or archaea of the same or multiple species, and by the specific extracellular matrix they produce. The extracellular polymeric matrix (EPS) has a distinctive composition. Besides proteins and carbohydrates, it includes also extracellular genomic DNA (eDNA), which plays several structural and nonstructural dynamic (control) roles (Steinberg and Kolodkin-Gal 2015). The EPS lacks long modular chains found in animals, such as collagen (Exposito and Lethias 2013).

In general, the life cycle of a biofilm includes three main phases: (1) attachment to a surface (when individual cells attach to a biotic or abiotic surface by means of adhesins, which production and secretion is triggered by the concentration of specific substances such as oxygen or sugars in the environment); (2) production and deposition of EPS, with consequent inhibition of flagellar movement, increase in the production of EPS by non-motile cells embedded in the matrix, and cell proliferation and differentiation; (3) dissolution of the matrix and disaggregation of the system with dispersion of cells (a regulatory response to the presence of high levels of toxic waste products, or to cell starvation in the innermost layers of the biofilm due to the growth of the system beyond its capabilities for the transport and distribution of nutrients). Some species such as *Mxyococcus xanthus* exhibit phenomena such as organized motility (pack hunting and rippling behavior) and fruit body formation, all enabled by intercellular interactions and by employing EPS (Muñoz-Dorado et al. 2016).

The cohesion of a biofilm as an integrated functional unit capable of collectively maintaining itself results from the action of several types of control constraints operating at different ranges. Short-range control is performed at the level of interactions between individual cells, while medium-range control is performed at the level of ensembles of cells. Short-range control is exerted locally. It includes bacterial conjugation—an interaction through which one bacterium transfers genetic material to another through direct contact. In case of nutritional stress, it might also include the direct exchange of enzymes responsible for the control of some physiological processes. The spatial proximity achieved within biofilms strongly favors this type of interaction between individual cells.

Medium-range control is performed by a combination of quorum sensing processes (QS) and the EPS. These control processes are responsible for the formation of the biofilm and its overall functioning as an organized whole. QS is a process in which individual bacteria, in the presence of some specific boundary conditions or perturbations, release molecules into their environment and in turn sense and respond to the concentration of these molecules in their environment. Responses are thus calibrated to the number of bacteria present. It is a collective control process. The individual bacteria are both the dynamic constraints synthesizing and releasing an autoinducer molecule into the environment, and the controlled systems, because their own gene expression is modified in response to the concentration of autoinducer interacting with their receptors. The result is the generation of gradients of collective activation through the diffusion of signaling molecules. QS performs medium-range control because it can affect a large number of cells in a given region of the biofilm.

QS can be used for multiple ends, also in combination with EPS. An example is the process of biofilm formation and integration of cells into a cohesive structure, which takes place through the control of motility. QS molecules such as AI-2 (autoinducer-2), can function as attractants of bacteria of the same or different species as the releasers, thus favoring aggregation (Laganenka et al. 2016). QS molecules such as AHLs (acyl-homoserine lactone autoinducers) participate in the control of genes involved in the synthesis and deposition of EPS molecules (Culler et al. 2018). Motility is directly modulated by the presence and type of EPS. During biofilm aggregation and formation, the rotation of bacterial flagella is mechanically blocked by the presence of EPS. This event triggers internal signal cascades that differentiate bacterial cells into persisters, characterized by an increased deposition of matrix molecules. This, in turn, inhibits the flagellar rotation in other cells and stimulates their matrix production, thus favoring the overall growth of the biofilm through a cascade effect. Inhibiting the motility of individual cells not only favors the growth of the system but prevents its disaggregation by avoiding the escape of motile cells. Moreover, inhibition of motility and production of different types of EPS structures favors the appearance of microconsortia by creating regions of space in which cells of different species of bacteria are grouped together, share a similar extracellular environment (for example an anaerobic one), and collaborate to perform certain functions (Flemming and Wingender 2010).^6^ EPS also controls direction of movement of cells within the biofilm, which may follow the orientation of eDNA fibers such as in *P. aeruginosa* (Steinberg and Kolodkin-Gal 2015). Or it can even control the collective movement of the whole system, such as in the case of *Mxyococcus xanthus*, in which the swarm follows matrix trails deposed by cells located at its edges (Muñoz-Dorado et al. 2016).

EPS structures are characterized by weak physicochemical interaction, by the entanglement of biopolymers, and by the storage of molecules, which can be released in the system. Matrix components interact so changing the activation state of EPS structures and proteins. Therefore, EPS does not only play the role of structural constraint. It is a dynamic structure which is sensitive to the mechanical and chemical state of the surrounding region of the system and changes its state accordingly. While doing so it operates as a medium-range controller. Variation in the EPS properties can control the production of its own components not only by blocking the rotation of flagella. In *B. subtilis* increased osmotic pressure due to changes in the EPS, activates KinD kinase in the membrane, which starts a signaling cascade leading to TasA amyloid fiber production. Increased production of matrix components is also controlled by direct binding of EPS to EpsAB membrane receptor (Steinberg and Kolodkin-Gal 2015). Moreover, changes in the matrix can lead to the retention or release of extracellular enzymes, directly or through vesicles, and to their activation or inhibition. Such enzymes participate in functional activities of the biofilm such as extracellular digestion and signaling. The same happens with sequestration, accumulation, and release of ions (Flemming et al. 2007). Depending on its state, the EPS selectively and dynamically constraints the physiology and behavior of large groups of cells and contributes to the development and functioning of the system (Flemming and Wingender 2010).

Intercellular and extracellular control is exerted in the biofilm at short and medium ranges upon ensembles of components. It is responsible for biofilm’s cohesiveness and the coordination of the activity of its parts—both cells and extracellular structures. However, the degree of integration in biofilms is limited to local interactions and medium-range gradients. Regions of functional cohesiveness enabled by differences in the EPS properties are not in turn coordinated at the system level. One reason is the lack of long-range control relative to the size of the system.

I will return on the limits of integration based on short- and middle-range control in biofilms and discuss their implications for individuality after showing that long-range control is possible in animals and what role it plays in integrating their physiology.

## Animals

Animals perform control at short, medium, and long ranges. The first two types of control exhibit general similarities with what was discussed with regards to biofilms. As animals mainly differ in that they employ also long-range control, I will focus more on the latter. But let us proceed in steps.^7^

Short-range control is exerted locally between individual cells. An example can be found during cell differentiation at the early stages of the development of the well-studied sea urchin *Strongylocentrotus purpuratus*. One mode of control at work during differentiation and separation between mesoderm and endoderm cells relies on the exchange of activation and inhibition signals between adjacent cells (Arnellos et al. 2014). Interestingly, this local control, combined with larger scale spatial cues, can have a medium-range cascade effect forming two distinct gradients of activation that lead to two different groups of cells. However, medium range control such as exerted by the ECM has often priority over cell-to-cell interactions in determining cell fate (differentiation) and behavior (Guilak et al. 2009).

A variety of medium-range controls are responsible for integration at the level of groups of cells or tissues. Compared to biofilms, animals are distinguished by a higher differentiation and modularity and more specific control, often associated with distinct spatial regions. For example, ECM structures interact with cells in assembling (or disassembling) supra-cellular structures. These give rise to boundaries and interfaces such as the basement membrane of epithelial tissues (Nistico et al. 2012). A type of medium-range control taking place in differentiated regions or subregions is mechanotransduction, where a change in the mechanical forces exerted by the ECM controls the fate or the activity of groups of cells by activating gene transcription. This triggers cell differentiation, cell division or modulates specific behaviors (Halder et al. 2012). In tissues, fine-grained control is performed for example by resident immune cells, which play several regulatory and coordinating roles by moving through the ECM fibers and delivering highly specific signals (Lee et al. 2015). In a nutshell, medium-range control in animals dynamically constraints the physiology and behavior of large groups of cells, contributes to the spatial localization, differentiation, and stabilization of specific cell types in distinct tissues, and it integrates and coordinates their activities towards physiological goals.

Medium-range control plays a decisive role in organogenesis during development, often involving interactions between two macroscopic structures, the parenchyma, made of layers of cells, and the stroma, which is composed of cells, vessels, and ECM. As discussed by Montévil and Soto (2023), in the development of the mammary gland, the epithelial cells of the parenchyma proliferate spontaneously unless constrained.^8^ Proliferation is constrained by the stroma containing ECM and connective tissue cells. The motility of the epithelial cells is also constrained by the structure of the ECM fibers to which they can attach and that they can pull in order to move, by the size of the pores in the matrix and by the rigidity of it. The structure of the ECM as well as the adhesion with other cells may facilitate or inhibit movement and determine the type of proliferation. A globular structure of the ECM, for example, does not allow cells to attach and move. In this scenario, cell proliferation gives rise to a sphere with a central lumen, the acinus. A fibrillar matrix, instead, allows for movement and proliferation of groups of cells in a given direction determined by the orientation of the fibers. In this case cells elongate and give rise to structures akin to ducts.

Long-range control is realized in animals mainly by means of vascularization and by the nervous system. Vascularization is the simplest way to exert control at larger scales by making components mobile, and thus allowing control molecules and cells to diffuse in a fluid medium in a constrained manner. In such a way they can reach areas that would be impossible or extremely improbable to reach – and to do so in the right concentration – through diffusion alone. Moreover, vascularization allows for their distribution throughout the system. It is much more efficient than unconstrained diffusion and can in principle reach all parts of the system.

Pervasive vascular systems are widespread in animals and are not unique to vertebrates. Echinoderms, for example, have a water vascular system, a network of fluid-filled canals generally including a ring canal, radiant canals that extend from it and from which shorter lateral canals extend. They also have a basic circulatory (*haemal*) system with a central ring and five radial vessels. Many chemical messengers necessary for metabolism, development and reproductive functions are transported directly within the body through these systems, while the movement of other messengers still relies on diffusion although within a constrained space, the perivisceral coelom: a fluid-filled cavity in which the major organs are suspended (Wasson and Watts 2007).

Vascularization allows for endocrine control by means of transport and distribution of hormones. Hormones are effector molecules produced and released by cells in glands under a variety of physiological and environmental conditions. They are transported by circulatory systems to distant tissues and organs to control their activities. In such a way they can affect groups of distant cells. They bind to specific receptors on the surface of cell membranes or in the case of steroid hormones, which can move through cell membranes, directly within cells. By interacting with cells, they modulate their activities by modifying the operations of existing proteins or by triggering gene expression. As a result, through hormones animals can control physiological activities taking place in organs and tissues and related to metabolism, growth, development, bodily fluid composition, behavior, reproduction etc. (Hiller-Sturmhöfel and Bartke 1998). One example of control by hormones is insulin. In mammals, depending on the concentration of glucose in the blood and on signals from the guts and the brain, insulin is released in high quantity in the blood by pancreatic beta-cells (Röder et al. 2016). Pancreatic beta-cells and the insulin they secrete are regulatory control constraints that modulate, among other things: the uptake of glucose in muscle and adipose tissue, glycogenesis in the liver, muscle, and fat cells, and (by inhibition) glucagon secretion by pancreatic alpha-cells^9^. Hormones can also lead cells to modify the organization of the ECM in which they are embedded. The modified ECM, in turn, (as a medium range controller) has decisive effects on the physiology and organization of the tissue. During the development of the mammary gland, for example, by inducing modification of the ECM, mammotropic hormones (estradiol, progesterone, prolactin) are involved in the generation of distinct patterns of organization of epithelial cells and the consequent production of different structures (Montévil and Soto 2023).

Long-range control is also realized through the nervous system by means of signal transmission architectures realized by networks of neurons.^10^ Neurons effectively extend long-range control by more rapidly transmitting a signal and by adding the capacity to target specific locations which may be far from the origin of the signal. The two major hypotheses on the origin of the nervous system, i.e., the contractile network hypothesis and the secretory network hypothesis, both characterize the emergence of the early nervous system as a response to the need to establish forms of thorough control upon tissues and organs (movement and integration of signals) in organisms with increasingly larger bodies (Arendt 2021). They emphasize the importance of the integrating function of early nervous systems at long ranges over the cognitive function, and the continuity of physiological control functions across different types of biological subsystems.

In cnidaria, the nervous system operates, and it is thought to have originated according to the contractile network hypothesis, as a means to coordinate muscle contraction for movement (Keijzer et al. 2013) and for the transport of food in the guts (Furness and Stebbing 2018). In jellyfish individual muscles can communicate directly with adjacent ones, but a synchronized propulsion movement requires a fast long-range coordination of their contractions. This is achieved by neurons, which control the contractions of muscle cells across whole sheets of muscles. The contractions of the muscles responsible for the movement of food in the guts are similarly controlled. The enteric nervous system of mammals controls a wide range of movements of the gut muscles, including peristaltic movement, segmentation or nonpropulsive mixing, slow orthograde propulsion, and retropulsion of noxious substances (Fleming et al. 2020). It also controls a variety of other activities, such as blood flow through the mucosa, fluid balance between the intestines and fluid compartments of the body, gastrointestinal hormones, secretion of gastric acid, transport of nutrients, etc. (Furness 2015). One hypothesis for the origin of the enteric nervous system is that it is the descendent of the first brain which emerged in hydra (also part of the phylum of cnidaria), an animal with a tubular body open to water from outside (Furness and Stebbing 2018). A network of several hundred neurons residing in the wall of the gut tube controls the contractions of the layers of longitudinal and circular muscles that are responsible for the peristaltic movements that propel and mix food through the gut.

The secretory network hypothesis on the origin of the nervous system focuses on chemical wiring instead, and it identifies the early function of the nervous system in maintaining and enhancing signaling efficiency to reduce the inefficiency of chemical signaling taking place through diffusion in the larger bodies of animals (Arendt 2021). According to this hypothesis, ancestors of neural cells were ciliated cells that started secreting neuropeptides. They specialized in cells capable of sensory perception and neuropeptide release. Scattered among other cells, these specialized sensory-effector cells enabled synchronized activity (pulses or waves of activities of ensembles of cells) and the integration of signals by linking up into a chemical network capable to ensure the release of peptides across entire fields of cells with a more coherent effect (Jékely 2021). To overcome the limitations of the exchange of neuropeptides by diffusion, they may have developed projections from cellular elongations containing secretory vesicles that increased the total membrane surface available for secretion and formed synaptically connected nerve nets. This hypothesis identifies a continuity with the current nervous systems, which are still largely chemically wired (Bechtel and Huang 2022), and with neuroendocrine control.

Neuroendocrine control is a form of long-range control that relies on both the neural and vascular systems to exert integrated control on large parts of the body. In vertebrates, the bridge between these two systems, vascular and nervous, is constituted by the hypothalamus, by means of which the nervous system extends and coordinates endocrine controls and the release and distribution of hormones in the circulatory system (Leng 2018). The hypothalamus integrates the states of different physiological activities and variables with the modulation of behavioral, autonomic, neuroendcorine, and motor subsystems. It controls several fundamental physiological functions: blood pressure and electrolyte composition; energy metabolism; reproductive behaviors; body temperature; defensive behavior; sleep-wake cycle (Kandel et al. 2021). In particular, the neuroendocrine activity of the hypothalamus controls the secretion of hormones by the pituary gland. The posterior pituary contains hormone-secreting terminals of the hypothalamic region, which secrete hormones such as vasopressin and oxytocin hormones in the circulatory system^11^. The anterior pituary contains instead endocryne cells, control constraints which secrete a vast array of hormones in the blood.^12^ In turn, these secretory activities of the endocryne cells are controlled by stimulatory and inhibitory factors, such as peptides and dopamine, released by the hypothalamus into a specialized circulatory system that connects with the anterior pituary.

In sum, examples from animals show that long-range control makes possible the communication between distant parts of a multicellular system, and consequently the control of different tissues and large ensembles of cells.^13^ Compared to medium-range control, long-range control makes possible not only the coordination of the activity of parts to realize a physiological function localized in a specific region or tissue, but the coordination of different physiological activities across the whole system. In doing so, it allows for a high degree of integration by making mutually dependent for their activities and existence different components that would not otherwise interact, and it canalizes their effects towards the maintenance of the organism.

## Physiological Integration and Degrees of Individuality

Biological control is performed by dynamic constraints that modulate the behavior of other components. It is responsible for the coordination of the activities of the components of a biological system, so that they take place when and in such a way that they contribute to the maintenance of the system. Control can be exerted in different ways. With a focus on multicellular systems, it is useful to look at differences of control in terms of range, to show how different parts and their activities can be modulated in increasingly bigger systems. Short-range control mainly relies on cell-to-cell interactions. It may allow for the modulation of activities between individual cells with very localized effects, such as conjugation in bacteria or the exchange of enzymes. Medium-range control – such as exerted for example through QS, and by the EPS and the ECM – operates instead on ensembles of cells by dynamically constraining them. The differences between the role of QS and of the EPS in biofilms, and that of the ECM in animals show that the specificity of medium-range control can vary in different multicellular systems. The common feature, however, is that it may result in the coordination of the activities of ensembles of cells to realize a physiological function localized in a specific region or tissue. Finally, long-range control is exerted between distant parts of a multicellular system and can reach a larger number of them. As discussed in the previous section, vascularization can allow for hormones to reach cells situated throughout the body of an animal. The nervous system can increase the speed of control interactions and reach specific locations. Through these systems, long-range control makes possible the communication between distant regions and the coordination of their activities.

Biofilms, as discussed in Sect. 3, illustrate the limitations of integration based on short and medium range control that result from lack of long-range control. It is true that biofilms realize a basic form of vascularization through the organization of the hydrophobic molecules of the EPS into rudimentary channel structures, folds in the matrix that harness the flow of fluid and allow nutrients from the periphery to reach the bacteria residing in the inner region (Cairns et al. 2014). However, this rough vascular system has neither the same reach nor the same capability for controlling activities throughout all the regions of the system. Most transport and signal distribution in the system is carried out locally by diffusion, and this is shown by the limited growth in size and spatial differentiation of biofilms. This strongly limits the capabilities to coordinate activities across the system. Moreover, as argued by (Militello et al. 2021), the limitations of biofilms integration may derive also from the lack of a cohesive and dynamic collective border. Finally, another aspect which limits the degree of integration in biofilms is the type of medium-range control exerted by QS and EPS. It is coarse-grained control that lacks the specificity and the capability to target specific parts that is instead exhibited by the examples of medium-range controls in animals. Control by QS signal molecules as well EPS enzymes and ions relies on diffusion and gradients of concentrations rather than fine-grained interactions. Moreover, the EPS lacks sharp modularity and its mechanical control capabilities mostly depend on the variation of the amount of matrix produced.

Unlike what happens in systems that rely only on short and medium-range control, long-range control provides a multicellular system with the capability to connect several distant parts of the body. This makes it possible to modify the activities of those parts on the basis of the status of several physiological factors to which these controlled parts are not necessarily sensitive or from which are not directly affected. Moreover, by reaching distant parts of the body, long-range control coordinates not only ensembles of cells towards one physiological activity, like medium-range control does, but also different physiological activities across the whole multicellular system.

This analysis shows that while control in general is a decisive element for physiological integration, different ways to realize control can contribute to achieve integration in different ways. More precisely, it shows that the range of control is, at least, one of the factors responsible for the *type* and *degree* of physiological integration of multicellular systems. The parts of different self-maintaining multicellular organizations, from biofilms to animals, are mutually dependent for their production, maintenance and activity. However, this dependence can be achieved by controlling the parts through chains of local interactions (short-range control), interactions with ensembles of cells (medium-range control), and across the whole system (long-range control). On this view, each new range of control allows for a higher degree of integration. It gradually extends the possibility of coordinating larger numbers of increasingly distant parts. Consequently, it increases the capability of a system to modify itself on the basis of its internal state and that of the environment as one cohesive entity, in which the behaviors of parts, ensembles of parts, tissues and organs, and whole physiological activities are made compatible and collectively contribute to the maintenance of the system.

Understanding how physiological integration is achieved and the fact that it comes in degrees, are particularly relevant for the debate on biological individuality. Accounts of individuality often refer to physiological integration as a source of cohesion: one of the criteria that may allow us to identify individuals, distinguish them from their background and other entities, to count, compare and track them. Kaiser and Trappes have recently identified and discussed the general problem agenda of biological individuality, as consisting of six main interconnected questions (Kaiser and Trappes 2021). Integration is included in this list as a question of unity, that is of what binds together the parts of a biological individual. The other questions concern identification, demarcation, parthood, uniqueness and temporality. Integration figures in Godfrey-Smith’s account of evolutionary individuality together with bottleneck and reproductive specialization among the three dimensions that can be used to distinguish between reproductive biological systems (Godfrey-Smith 2009, 2013). Integration is characterized in term of the mutual dependence, or indivisibility, between parts and division of labor. Other accounts refer to integration in terms of unity and autonomy (Santelices 1999), or of the combination between high cooperation and low conflict between the parts of a biological system (Queller and Strassmann 2009).

Integration is a core notion for biological individuality in general, but it is especially central for those non-evolutionary accounts of biological individuality focused on the physiology of biological systems, that is, on how parts and their activities are kept together. Given the importance of integration for biological individuality, it is necessary to provide precise accounts of integration that are more detailed than the general idea of mutual dependence between parts. This is particularly relevant for an account of individuality such as the physiological one, which has not received enough theoretical treatment and, unlike evolutionary individuality, it has not given rise to precise and detailed analyses. Mutual dependence may be achieved by very different systems so that conceptual tools are needed to understand these differences, evaluate their implications and discuss whether and how these systems can be considered biological individuals in a physiological sense.

The organizational framework focused on control provided in this paper is a possible way to develop the notion of physiological individuality in the details beyond the general notion of mutual dependence, by focusing on the ways in which the parts of a multicellular system are bound together to achieve integration. If one considers Kaiser and Trappes’s (2021) problem agenda for biological individuality from the point of view of physiology, answering the question of unity is central to address the others. It is the understanding of how parts are bound together in the physiology of a multicellular system that can allow answering questions about what a part is: let us think for example at the discussion in Sect. 2 about the need for multicellular control to achieve physiological integration and the importance of identifying both cells and the extracellular matrix as the main controllers. The same applies to questions of identification and demarcation, as what allows to identify a physiological individual and distinguish it from a background may be a specific network of production and control relations. The question of uniqueness can be addressed in terms of different realizations of a given type of organization. As discussed in this paper, different forms of control can be examples of properties that make different physiological individuals unique. Regarding temporality, starting from a framework focused on unity in physiology, advocates of the organizational account have addressed questions of diachronic identity and temporal stability of biological systems. They have explained it in terms of their organizational invariance, that is the maintenance of a basic network of interactions despite the constant turnover of the parts that realize it (Di Frisco and Mossio 2020; Mossio et al. 2016).

Moreover, by making the notion of integration more precise and by looking at organization, this approach can address not only general questions on individuality such as those identified by Kaiser and Trappes (2021). It can also provide support or theoretical grounding for other accounts of individuality based on specific attributes. For example, the ideas of cooperation and low conflict between parts employed by Queller and Strassmann (2009) can be grounded in the notions of physiological control and integration in self-maintaining multicellular organizations.

An important implication of this framework, which bases individuality in integration through control, is that by distinguishing degrees of physiological integration, it supports the thesis that there are different ways to achieve individuality and that individuality comes in degrees. The idea that there are different kinds of individuals depending on whether or not they exhibit certain properties, was for example advanced by Santelices. He lists three different classes of attributes that allow to distinguish between kinds of individuals by means of the presence or absence of genetic uniqueness, of genetic homogeneity, and of autonomy and physiological unity (Santelices 1999). This results in eight different combinations of attributes, each defining a kind of individual. Others, instead, focus on degrees rather than types of individuality. They include some of the accounts mentioned in the previous paragraphs (Godfrey-Smith 2013; Kaiser and Trappes 2021; Queller and Strassmann 2009), and others such as, for example, Krakauer’s idea of informational individuality based on degrees of environmental dependence and inherited information (Krakauer et al. 2020), and Griesemer’s proposal that the degree of individuality changes during development (Griesemer 2018). These accounts describe individuality as a continuum in a space whose different dimensions correspond to different attributes associated with individuality. Different instances of biological individuality would be distributed in this space according to the degree to which they exhibit a specific set of properties: from weaker to stronger individuals.

According to the framework developed in this paper the degree of individuality of a biological system is determined by the ranges of control performed within it. On this account, biofilms are characterized as integrated systems, therefore physiological individuals^14^, but not with the same degree of individuality as animals, because although they employ short and medium-range control, they lack long-range control. Unlike some of the other gradualist accounts of individuality, this account, as discussed in this paper, is unidimensional, with control range as the main variable property. However, this is just a first step. It cannot be excluded that different dimensions of control can be identified besides range – for example the specificity or precision of control interactions or the degree of crosstalk between different control systems – so that a more complex view can be provided in the future.

## Conclusions

This paper has given an account of physiological integration as resulting from how the activities of the components of a biological system are controlled and coordinated so that they can contribute to the maintenance of the system. In multicellular systems, control is performed by cells and extracellular structures. By analyzing examples of different types and ranges of control in biofilms and animals, it has been argued that the most cohesive form of integration is achieved when different physiological activities are coordinated at the system level. Such coordination is realized through long-range control.

Different ways of realizing control, thus, can account for differences of integration between multicellular organizations. The difference between integration in biofilms and animals largely depends on the lack of capability of biofilms to realize long-range control, which in animals is enabled by vascularization and by the nervous system. A further factor to consider is the fact that biofilms realize a coarse-grained distributed control based on gradients of concentrations lacking specificity and modularity.

On this account, physiological integration comes in degrees. Given the role played by integration in current accounts of biological individuality, an implication of this perspective is that the physiological dimension of individuals can be given a precise characterization in terms of control, and that their degree of individuality depends on the ranges at which they exert control on their parts so as to coordinate their activities. For example, insofar as biofilms lack long-range control and are less integrated than other multicellular systems, they can be considered also as weaker physiological individuals. The same tools could in principle be applied to evaluate the degree of individuality for other forms of multicellular organizations.

## Footnotes

1 Multicellularity may have originated more times, and will probably do it again, if we take into accounts also examples of multicellular organizations evolved *de novo* in the laboratory from previously unicellular organisms, such as the recent case of the yeast *Saccharomyces cerevisiae* (Bozdag et al. 2023).

2 It is important to clarify that ranges of control are considered here in terms of differences in types of interactions and effects: on single cells, on groups of contiguous cells, or on cells or groups of them which are located in different, non-contiguous parts of the system. These distinctions are aimed at identifying qualitative break points in control relationships, not at measuring absolute distances.

3 For the integrative role of control see for example work on the integration of biological control mechanisms in biological organizations by Bich and Bechtel (2022), and Weber’s notion of ‘coherent causal control’ (Weber 2022), a form of control which causes a change in the distribution of values of the downstream variables from some initial state to a coherent state that supports some coordinated biological activity. This paper adopts an approach closer to the former (mechanistic) account, which is more fine-grained and includes the requirement that control plays a regulatory function within the organism by contributing to its maintenance (see Weber 2022 for a discussion of this difference between the two approaches).

4 The basic idea of mutual dependence between biological constraints, or ‘closure of constraints’ (Montévil and Mossio 2015), does not account for how living systems can change, that is, modify their own organization or internal dynamics in time (Bich and Bechtel 2022). In the organizational framework, physiological and developmental change are accounted for in terms of regulatory control (Bich et al. 2016; Bich and Skillings 2023). Regarding how the organizational framework takes into consideration change on a larger, intergenerational scale through the notion of historicity, see Montévil and Mossio (2020).

5 According to most recent characterizations by the organizational account, biological agency can be defined as the set of activities of a living system, modulated by regulatory control, that modify the environment of the system and are performed in such a way as to contribute to the maintenance of the system itself (Moreno 2018).

6 By doing so, medium-range control favours the establishment of forms of short-range control. In this respect, short-range control is not as much an integrating factor as instead a consequence of some degree of integration, spatial in this case, already in place.

7 I will consider here the minimal multicellular organization realized by a set of mutually dependent constraints (or ‘closure of constraint’) subject to regulatory control. However, it is important to point out that the microbiome also plays an important role in the physiology of animal hosts. Considering the interactions between host (the multicellular organization considered here) and its microbiome is beyond the scope of this paper, which aims to provide a basic general framework. It would require focusing on a larger entity and evaluate case by case whether and why some interactions give rise to a higher-order integrated organization including both systems. Some preliminary work in this direction has been done by Bich (2019) and by Skillings (2019).

8 To analyze the specific role of different tissue components and structures in organogenesis, Montévil and Soto (2023) rely on simplified 3D culture systems and computational models of ex vivo development of organs.

9 For a detailed analysis of this type of control in terms of constraints, see (Bich et al. 2020). On their account, the constraint controlled by insulin for glucose uptake is the transport/hexokinase (GT/HK) subsystem of the cells of muscle, adipose and liver tissues, which facilitates the migration of the glucose transporter GLUT4 to the plasma membrane so as to allow the phosphorylation of glucose into G6P and its transport within these cells. In the case of glycogenesis in liver cells, the constraints controlled by insulin are the enzymes glycogen synthase (GSase) which are responsible for the production of glycogen from G6P. For the inhibition of glucagon secretion by pancreatic alpha-cells, the controlled constraint is the membrane potential of alpha-cells.

10 This discussion focuses on the role of the nervous system in the control and integration of physiological activities. Other work within the organizational framework has addressed instead the integrating role of the nervous system in relation to multicellular agency (see for example Arnellos and Moreno 2015; Keijzer and Arnellos 2017). Physiology and agency represent two different foci on multicellular systems. Physiology considers how parts are integrated so that they can perform all the activities necessary to maintain the system. Agency considers how such a system can recruit some of these integrated activities to interact with the surroundings to keep viable in its environment by procuring nutrients, escaping from predators, etc. The two perspectives do not fully coincide. While multicellular agency requires an integrated physiology—otherwise there would simply be no system—physiological integration in principle would not require the system to be an integrated, unified or centralized agent, as agential capacities can be also exerted collectively by the different components of the system (let us think for example of highly modular systems such as some plants might be according to Arnellos and Moreno 2015).

11 Among other things, vasopressin is involved in the control of blood pressure and water concentration, while oxytocine in the control of uterine smooth muscle and of milk release (see Leng 2018, for a thorough discussion of these hypothalamic hormones and their multiple roles).

12 They secrete hormones such as thyrotropin (TSH), prolactin (PRL), adrenocorticotropin (ACTH), beta-lipotropin, luteinizing hormone (LH), follicle-stimulating hormone (FSH), growth hormone (GH), prolactin. Their stimulation or inhibition is controlled, in turn, by a variety of hypothalamic substances (Kandel et al. 2021).

13 The absence or presence of long-range control may be linked to the different types of developmental processes that characterize systems like biofilms—which depend, in part, from the aggregation of previously independent parts—and animals—in which the different subsystems are much more intertwined from the very beginning of development. I thank an anonymous reviewer for raising this point. While addressing development is beyond the scope of this paper, it is nevertheless one of the natural directions for further work on integration.

14 The debate on biofilm individuality has mainly focused on them as possible evolutionary individuals, and whether they are capable or not of reproduction and of unitary interaction with their environment (Doolittle 2013; Ereshefsky and Pedroso 2013; Clarke 2016). However, considerations on how parts interact have played a role in Clarke’s rebuttal of the idea of biofilms as evolutionary individuals, specifically the fact that most interactions take place on too small spatial scales compared to the size of the biofilm to allow to consider them as reproducing and interacting as wholes.
